# Patient Perspective on Robotic-Assisted Total Joint Arthroplasty

**DOI:** 10.1016/j.artd.2024.101598

**Published:** 2024-12-20

**Authors:** Siddhartha Dandamudi, Kyleen Jan, Madelyn Malvitz, Anne DeBenedetti, Omar Behery, Brett R. Levine

**Affiliations:** a Department of Orthopaedics, Rush University Medical Center, Chicago, IL, USA; b Georgetown University School of Medicine, MedStar Georgetown University Hospital, Washington, D.C, USA

**Keywords:** Arthroplasty, Robotic surgery, Patient expectations

## Abstract

**Background:**

Robotic-assisted total joint arthroplasty (TJA) has gained popularity in recent years. Despite mixed patient and surgeon perceptions, conflicting evidence regarding efficacy and cost-effectiveness in comparison to manual TJA exists. Patients' beliefs surrounding robotic-assisted TJA remain unclear. This study aims to assess patients' expectations on robotic technology in TJA.

**Methods:**

A 9-question survey assessing patient understanding and expectations of the use of robotics in TJA was distributed to preoperative and postoperative hip and knee patients of five surgeons at a high-volume academic center. Responses were descriptively analyzed.

**Results:**

A total of 498 responses were collected. Of all respondents, 69.1% are aware of robotic usage in TJA, 68.5% are interested but unsure of the benefits, and only 19.5% feel it is superior to manual surgery. Most patients did not consider robotic TJA as minimally invasive surgery, with 61.7% stating they are not the same. In addition, 52.3% were not comfortable with extra or longer incisions for robotic procedures. Regarding surgeon choice, 94.9% did not consider if the surgeon is able to perform robotic TJA, 74.4% wanted their surgeon proficient in manual TJA, and 72.4% felt that surgeons who use robotic technology are not more capable than manual surgeons.

**Conclusions:**

Awareness and curiosity of robotic-assisted TJA exists; however, most patients did not appear to acknowledge superiority or benefits over manual surgery. Furthermore, patients appear to prefer surgeon proficiency in manual techniques, which may influence training programs in the future. Surgeons should weigh patient goals, expectations, outcomes, and costs when choosing to perform robotic TJA.

## Introduction

Robotic-assisted total joint arthroplasty (TJA) has gained popularity in recent years, with a 601.2% increase in volume of robotic-assisted surgeries performed between 2015 and 2020 [1]. The efficacy and utility of robotic-assisted TJA has been controversial among surgeons since its recent inception [[1], [2], [3]]. Capital expenditures and a limited number of robots initially slowed the widespread use of this technology, and concerns regarding time consumption, cost-effectiveness, and healthcare burden remain debated to this day [[4], [5], [6]].

Evidence of the effectiveness of robotic-assisted TJA remains mixed in the literature; Alrajeb et al. [7] conducted a meta-analysis that illustrated robotic-assisted total knee arthroplasty (TKA) had superior mechanical and anatomical alignment of components but similar functional, clinical, and complications when compared to manual TKA. Other studies demonstrate similar findings, reporting that robotic-assisted TJA results in accurate and precise bone cuts, but no difference in functional outcomes. Furthermore, robotic surgery is not without risks; the procedure is often longer with added costs, longer incisions, and carries risks of infection and fracture from pin sites [3,[8], [9], [10], [11], [12], [13]]. Overall, the existing evidence primarily investigates outcomes and cost of robotic TJA, while patient preferences and understanding of the robotic TJA have been largely unstudied despite increasing industry and provider-driven marketing of robotic platforms. As such, an opportunity for investigation is warranted.

The purpose of this study was to assess expectations of patients regarding robotic TJA to better characterize patient understanding, perspective, and conceptions surrounding the procedure. We hypothesized that the majority of patients would not seek out surgeons for their ability to perform robotic TJA, would not be satisfied with requiring additional incisions, would be able to distinguish robotic vs minimally invasive TJA, and would value proficiency in manual TJA.

## Material and methods

Institutional review board approval was obtained for this single-center cross-sectional survey study. A nine-question survey was created by the senior authors with questions geared to assess patient perspectives regarding robotic surgery in TJA (Table 1). In this study, robotic surgery is defined as the use of robotic systems or devices to assist in the precision and alignment of TJA. This study does not specify a particular robot or device brand and was not intended to answer questions regarding navigation or other alternative alignment options. The questions were devised to assess different aspects of patients' attitudes toward robotic surgery. Questions on baseline awareness of robotic surgery and comparisons to minimally invasive surgery aimed to discern if patients were distinguishing the two different surgical modalities. A subset of the questions aimed to gather comfort level of patients with their surgeon, specifically if they sought a particular surgeon out for robotic surgery and if they believed robotic surgeons to be superior at the procedure. Finally, two questions assessed how patients felt about longer incisions in robotic surgery and overall thoughts regarding robotic surgery in comparison to manual instrumentation.

**Table 1:** Patient survey responses regarding robotics in hip and knee arthroplasty.

Question	Yes %	No %
Have you heard of robotic technology for use in hip and knee replacement surgeries?	68.3	31.7
Have you heard of minimally invasive surgery?	83.1	16.9
Did you seek out your surgeon because they perform robotic-assisted surgery?	2.3	97.7
Do you consider robotic surgery to be the same as minimally invasive surgery?	38.3	61.7
Would you be okay with the surgeon having only performed surgery robotically assisted (never trained with manual instruments)?	27.5	72.5
Do you feel that surgeons that use this technology are more capable than those that do not?	25.5	74.5
Are you comfortable with your surgeon needing extra incisions or a longer incision to have robotic surgery?	43.9	56.1
How do you feel about robotic surgery?^a^	-	-
Robotic surgery is the same or similar to minimally invasive surgery?^b^	45	55

a Answers were not “yes” or “no”: **17.8%** said it is superior to manual surgery, **13.1%** said it is no different than manual surgery, and **69.1%** said they were interested but unsure of the benefits.

b Answers represent true (Yes) and false (No) responses.

Surveys were distributed to patients considering TJA across five fellowship-trained adult reconstruction surgeons’ clinics over a 3-month period at a high-volume academic center. Among the five surgeons, two routinely use robotics for primary TJA. No patient-identifying information was collected. Categorical and binary responses to questions were collected from the completed surveys. A descriptive analysis of the proportion of responses for each question was performed, and qualitative data were presented as proportions of responses using Microsoft Excel (Version 16.85, Microsoft, Redmond, WA).

## Results

A total of 498 patient responses were collected. Frequency and proportions of responses to the survey questions are shown in Table 1. More than two-thirds (68.3%) of respondents have heard of robotic surgery. In discerning patient perception between minimally invasive TJA and robotic TJA technique, 83.1% of patients have heard of minimally invasive surgery, and 61.7% do not believe robotic surgery and minimally invasive surgery refer to the same techniques. Nearly all respondents (97.7%) of patients did not seek out surgeons due to their ability to perform robotic TJA. Of the total, 72.5% responded that they would not be satisfied with surgeons who are solely trained in robotic TJA, and 74.5% do not consider surgeons more capable for ability to use robots. Regarding surgical incisions, 56.1% of patients answered that they would not be comfortable with longer incisions for robotic involvement in the procedure. Of the total, 17.8% of patients responded that robotic TJA was superior to manual surgery, 13.1% responded that it is equivalent to manual surgery, and 69.1% responded that they were interested but unsure of the benefits.

## Discussion

Despite increasing surgeon utilization, successful clinical outcomes, cost-effectiveness, and widespread industry and surgeon-directed marketing, patient perspectives regarding robotic TJA remained unclear and inadequately characterized at the time of this study [8,10,14]. Our data suggest that larger proportions of patients carry a degree of skepticism and curiosity rather than direct demand for robotic TJA.

Our data demonstrated that 68.3% of patients are at least aware of robotic surgery. This finding aligns with a study by Brinkman et al. [15] who performed a Google trends analysis that demonstrated a significant linear increase in online searches for robotic hip arthroplasty, which was also significantly more than the online search volume for conventional arthroplasty. Despite this awareness, our data show that only 2.3% of patients sought out surgeons for their ability to perform robotic TJA. As such, while patients understand that robotic surgery is an option, this knowledge may not primarily drive their choice of surgeon.

Notably, more than half of patients (56.1%) were not comfortable with longer or additional incisions required for robotic surgery. Concomitantly, 45% of patients believe a minimally invasive surgical technique is synonymous with robotic surgery, highlighting a likely discrepancy in patient understanding about the procedure. This represents a topic that surgeons may choose to incorporate in preoperative education with their patients. Furthermore, of the patients who reported interest in robotic surgery, 69.1% were unsure of the benefits. Despite potential advantages of eliminating outliers in component position especially with low-volume surgeons, robotic surgery carries additional risks including potentially multiple or longer incisions, pin-site fractures, pin-site infections, soft-tissue injury, and excessive blood loss [13,[16], [17], [18], [19]]. Our data demonstrating the discrepancies in patient understanding of robotic surgery and its risks underscore the importance of discussion with patients during the informed consent process.

With growing interest among surgeons and patients in robotic-assisted TJA, the relevant risks and costs of technology should also be considered on a more macroscopic level. Longer operative times with robotic TJA with its associated cost and risks have been previously reported by Lonner et al. [20]. Rajan et al. [5] similarly described that institutional volume plays a role in the cost per case of robotic-assisted TKA, with acquisition of equipment and additional costs only financially economical if an institution performs over 250 surgeries annually. All new surgeons adopting robotic TKA face a learning curve with increased operative times until proficiency is achieved [21,22]. With potential for increased costs and risks in mind, surgeons should be mindful of making shared decisions with patients on whether to pursue robotic TJA [[23], [24], [25], [26], [27]]. In situations where patients are charged an additional fee for use of robots, they may consider this cost more closely with better understanding of the aforementioned risks associated with robotic TJA.

Importantly, the impact on proficiency of fellow and resident trainees is a critical consideration in the era of increasing robotic TJA utilization. Duensing et al. [28] assessed resident preferences regarding training of TJA with robots and manual instrumentation, reporting decreased comfort performing manual TKA with high robotic case volumes during residency. With our study data demonstrating a preference among patients that surgeons should be trained with manual instrumentation, it is important to consider how robotic vs manual TJA should be handled in training. What are the implications of reducing the number of manual cases a resident will see during training? Will the next generation of adult reconstruction specialists feel as comfortable performing cases that require manual instrumentation? Such consideration and discussion should take place among training programs to assure that future surgeons can meet and exceed expectations of our patients.

This study is not without limitations. First, all data were collected from a single urban academic center, limiting its external validity for different regions or healthcare settings where robotic surgery is not as widespread or available. In densely populated regions, patients may have access to a larger number of surgeons offering robotic-assisted TJA and be more influenced by industry and provider-driven marketing campaigns, which can shape patient perceptions and expectations. Conversely, patients in less densely populated areas with limited access to robotic surgery may have different views. Second, the study did not collect demographic data, and thus cannot evaluate the response rate to the survey and whether there are any patient-related factors that influence responses regarding robotic surgery. Also, the survey-based study design makes the results prone to nonresponse bias; however, we believe that collecting nearly 500 responses helps minimize this effect.

## Conclusions

Although there is curiosity surrounding the use of robotic-assisted TJAs, most patients in this study do not believe there to be increased benefit compared to manual surgery. To create a care plan best suited for the patient, surgeons should discuss patient goals, cost, and long-term expectations and educate patients regarding benefits and risks when choosing to perform robotic TJA.

## Conflicts of interest

A.D. is a paid consultant for JointMedica Ltd. O.B. is a paid consultant for Medacta USA, Onkos Surgical, Enovis/DJO, and Signature Orthopaedics. B.R.L. receives royalties from Link Orthopedics; is a paid consultant for Enovis, Link Orthopedics, Merete, and Zimmer Biomet; receives research support from Zimmer Biomet; is in the editorial/governing board of *Arthroplasty Today*, Elsevier, Human Kinetics, SLACK Inc, and Wolters Kluwer Health; and is a board member of AAOS, American Association of Hip and Knee Surgeons, Knee Society, and MAOA. The remaining authors declare no conflicts to disclose.

For full disclosure statements, refer to https://doi.org/10.1016/j.artd.2024.101598.

## CRediT authorship contribution statement

**Siddhartha Dandamudi:** Writing – review & editing, Writing – original draft, Visualization, Validation, Project administration, Methodology, Investigation, Formal analysis, Data curation, Conceptualization. **Kyleen Jan:** Writing – review & editing, Writing – original draft, Visualization, Software, Methodology, Investigation, Formal analysis, Data curation, Conceptualization. **Madelyn Malvitz:** Writing – review & editing, Writing – original draft, Visualization, Methodology, Investigation, Data curation, Conceptualization. **Anne DeBenedetti:** Writing – review & editing, Writing – original draft, Project administration, Methodology, Investigation, Data curation, Conceptualization. **Omar Behery:** Writing – review & editing, Writing – original draft, Validation, Supervision, Project administration, Methodology, Investigation, Formal analysis, Conceptualization. **Brett R. Levine:** Writing – review & editing, Writing – original draft, Visualization, Validation, Supervision, Resources, Project administration, Methodology, Investigation, Data curation, Conceptualization.

## Appendix A Supplementary data

Conflict of Interest Statement for DeBenedetti

Conflict of Interest Statement for Behery

Conflict of Interest Statement for Malvitz

Conflict of Interest Statement for Levine

Conflict of Interest Statement for Jan

Conflict of Interest Statement for Dandamudi
