# Supplementary material for: Patient Perspective on Robotic-Assisted Total Joint Arthroplasty
Source: Arthroplast Today. 2024 Dec 20;31:101598. doi: 10.1016/j.artd.2024.101598 (PMC11731747; doi:10.1016/j.artd.2024.101598)
Supplement: Conflict of Interest Statement for Jan [file mmc5.pdf]

# CONFLICT OF INTEREST STATEMENT

## *American Association of Hip and Knee Surgeons*

(Adopted from the American Academy of Orthopaedic Surgeons disclosure statement)

Patient Perspective on Robotic Assisted Total Joint Arthroplasty

Manuscript Title

1. Royalties from a company or supplier (The following conflicts were disclosed)
   *None*

2. Speakers bureau/paid presentations for a company or supplier (The following conflicts were disclosed)

   *None*

3A. Paid employee for a company or supplier (The following conflicts were disclosed)

   *None*

3B. Paid consultant for a company or supplier (The following conflicts were disclosed)

   *None*

3C. Unpaid consultants for a company or supplier (The following conflicts were disclosed)

   *None*

4. Stock or stock options in a company or supplier (The following conflicts were disclosed)

   *None*

5. Research support from a company or supplier as a Principal Investigator (The following conflicts were disclosed)

   *None*

6. Other financial or material support from a company or supplier (The following conflicts were disclosed)

   *None*

7. Royalties, financial or material support from publishers (The following conflicts were disclosed)
   *None*

8. Medical/Orthopaedic publications editorial/governing board (The following conflicts were disclosed)

   *None*

9. Board member/committee appointments for a society (The following conflicts were disclosed)

   *None*

**Each author must sign AND print or type his/her name, date and submit a separate form**

In addition, one BLINDED Conflict of Interest form (no author names used) should be submitted per manuscript with all author disclosures.

| Kyleen Jan | [signature] | 09/19/2024 |
|---|---|---|
| Author Name (Print or Type) | Author Signature | Date |
